# Valorization Potential of a Novel Bacterial Strain, *Bacillus altitudinis* RSP75, towards Lignocellulose Bioconversion: An Assessment of Symbiotic Bacteria from the Stored Grain Pest, *Tribolium castaneum*

**DOI:** 10.3390/microorganisms9091952

**Published:** 2021-09-14

**Authors:** Mudasir A. Dar, Neeraja P. Dhole, Rongrong Xie, Kiran D. Pawar, Kalim Ullah, Praveen Rahi, Radhakrishna S. Pandit, Jianzhong Sun

**Affiliations:** 1 Biofuels Institute, School of the Environment and Safety Engineering, Jiangsu University, Zhenjiang 212013, China or 5501900034@ujs.edu.cn (M.A.D.); rrxie@ujs.edu.cn (R.X.); 2 Department of Zoology, Savitribai Phule Pune University, Ganeshkhind, Pune 411007, India; neerajadhole420@gmail.com; 3 School of Nanoscience and Biotechnology, Shivaji University, Vidyanagar, Kolhapur 416004, India; kdp.snst@unishivaji.ac.in; 4 School of Medicine, Jiangsu University, Zhenjiang 212013, China; kalimbtk@gmail.com; 5 National Centre for Microbial Research, Trinity Complex, Pashan, Pune 411021, India; pravin@nccs.res.in

**Keywords:** red flour beetle, gut system, cellulose degradation bacteria, lignocellulose, hydrolyzed biomass, bioethanol

## Abstract

Bioconversion of lignocellulose into renewable energy and commodity products faces a major obstacle of inefficient saccharification due to its recalcitrant structure. In nature, lignocellulose is efficiently degraded by some insects, including termites and beetles, potentially due to the contribution from symbiotic gut bacteria. To this end, the presented investigation reports the isolation and characterization of cellulolytic bacteria from the gut system of red flour beetle, *Tribolium castaneum*. Out of the 15 isolated bacteria, strain RSP75 showed the highest cellulolytic activities by forming a clearance zone of 28 mm in diameter with a hydrolytic capacity of ~4.7. The MALDI-TOF biotyping and 16S rRNA gene sequencing revealed that the strain RSP75 belongs to *Bacillus altitudinis*. Among the tested enzymes, *B. altitudinis* RSP75 showed maximum activity of 63.2 IU/mL extract for xylanase followed by β-glucosidase (47.1 ± 3 IU/mL extract) which were manifold higher than previously reported activities. The highest substrate degradation was achieved with wheat husk and corn cob powder which accounted for 69.2% and 54.5%, respectively. The scanning electron microscopy showed adhesion of the bacterial cells with the substrate which was further substantiated by FTIR analysis that depicted the absence of the characteristic cellulose bands at wave numbers 1247, 1375, and 1735 cm^−1^ due to hydrolysis by the bacterium. Furthermore, *B. altitudinis* RSP75 showed co-culturing competence with *Saccharomyces cerevisiae* for bioethanol production from lignocellulose as revealed by GC-MS analysis. The overall observations signify the gut of *T. castaneum* as a unique and impressive reservoir to prospect for lignocellulose-degrading bacteria that can have many biotechnological applications, including biofuels and biorefinery.

## 1. Introduction

The rapid depletion of fossil fuels and increased emission of greenhouse gases are the major issues of modern civilization that demand immediate addressal. Consequently, these issues have attracted much attention from scientists and academicians as well as policy makers all over the world. Therefore, in recent decades, more focus has been given to prioritizing the production of biofuels from renewable and sustainable resources like lignocellulose. Lignocellulosic (LC) plant matter is the most abundant and renewable energy resource that originates from agriculture, industry, municipality, and forestry [1]. Every year, agricultural practices alone contribute millions of tons of solid wastes in the form of rice straw, wheat straw, corn stover (CS), sugarcane bagasse (SCB), etc. [2]. LC-based agro-waste is mostly composed of three polymers, cellulose, hemicellulose, and lignin, among which cellulose and hemicellulose represent a major reservoir of fermentable sugars. Using advanced technologies, this promising, low-cost, and renewable energy resource can be transformed into a variety of useful products such as amino acids, sugars, biofuels, etc.

The biological hydrolysis rendered by some enzymes is foreseen as a promising strategy for bioconversion and utilization of LC biomass [3] because it offers pure and sustainable by-products [4,5]. Due to the recalcitrant and heterogeneous nature of LC, the biological conversion relies on multiple enzymes such as cellulases, xylanases, and laccases [6]. However, cellulases and xylanases are the key enzymes that can be employed for the bioconversion of LC biomass into useful products. In nature, these enzymes are secreted by some natural biomass utilization systems (NBUSs) such as bacteria, fungi, insects, annelids, mollusks, and nematodes [7,8,9]. The underlying mechanisms by which these NBUSs degrade cellulose could potentially valorize the LC biomass into commodity chemicals [10]. Among the NBUSs, insects have evolved the most efficient and sophisticated bioconversion systems through synergism with symbiotic gut microbiota, particularly bacteria [11]. To date, a considerable diversity of cellulose-degrading bacteria has been explored from a variety of insects [12]. Although many cellulose-degrading microorganisms have been reported from different environments, the efficient and economical bioconversion of LC into monomer sugars still remains a challenge. Moreover, the current cellulolytic technologies are not sufficient for the production of 3rd generation bioethanol [13], thus prospection for more efficient bacterial strains is direly important to make biofuel production economical.

Being the most diverse and dominant class in the kingdom Animalia, Insecta possess superior adaptations to a variety of foods and environmental conditions. One such insect, the red flour beetle, *Tribolium castaneum* Herbst (Coleoptera: Tenebrionidae), is a stored grain pest with economic importance [14,15]. It attacks many important crops such as wheat, maize, peanuts, cashews, etc. [16,17], and causes qualitative and quantitative loss of grain productivity. Apart from cereals and grains, it predominantly feeds on wheat bran which is a rich source of fibrous polysaccharides such as xylan and cellulose [18]. Although the occurrence of endogenous cellulases in *T. castaneum* was reported recently [19,20,21,22], not much is known about the cellulolytic microbes inhabiting its gut system. Additionally, unlike other insects such as termites [23,24], Lepidoptera [12,25], orthopterans [26], and dung beetles [27,28], bacterial cellulases and their activities from the gut of *T. castaneum* have not been studied yet. Hence, the prospection of cellulolytic bacteria in *T. castaneum* would not only contribute to the basic understanding of host–microbe interactions but would also highlight its prospective applications for the discovery and bioengineering of potential bacteria and their use in biorefinery and pulp industry alongside biofuel production. Given this importance, the objectives of the present study were to explore the cellulolytic bacteria from the gut system of *T. castaneum*, then identify and screen them for cellulose degradation. Further, the cellulolytic potential of the most efficient bacterial strain was evaluated and characterized for the production of LC-hydrolyzing enzymes that can be employed to break down various agricultural wastes, such as SCB, corncob powder (CCP), wheat husk (WH), grass straw (GS), etc., into many commodity products such as reducing sugars, biofuels, etc. 

## 2. Materials and Methods

### 2.1. Reagents and Substrates

Agricultural wastes including corncobs, WH, GS, and SCB were acquired from the rural areas of the Pune district of Maharashtra, India. These substrates were pre-treated mildly with an alkaline solution (0.1 N) of sodium hydroxide (NaOH) for 24 h at room temperature. After pre-treatment, the substrates were prepared as described previously [29], while commercially available substrates such as carboxy methyl cellulose (CMC), microcrystalline cellulose (Avicel 101^®^), and Whatman^®^ filter paper grade 2 (FP) were procured from Sigma-Aldrich, (Millipore Sigma, St. Louis, MO, USA). The Berg’s minimal salt (BMS) media contained different salts in varying proportions as reported earlier [30].

### 2.2. Identification and Dissection of Insects

The insects collected from the wheat husk were identified based on their morphological characteristics [31], particularly head and thoracic regions, using interactive dichotomous keys from Lucid Professional version 2.0 [32] (https://lucidcentral.org/, accessed on 18 March 2021). Before dissection, the adult beetles were starved for 8 h followed by brief washes of ethanol grades 50% and 70% each for 30 s and lastly of absolute ethanol for 60 s. The insects (*n* = 20) were sacrificed under aseptic conditions and the revealed gut systems were weighed followed by homogenization in 0.4 mL phosphate-buffered saline (PBS, pH 7.0) by using a micropestle. The homogenized mixture was serially diluted up to 10^−8^ and spread on Luria–Bertani (LB) agar plates and BMS agar which contained 0.5% CMC as a screen to grow cellulose-degrading bacteria only. The BMS agar plates were incubated at 37 °C for 48 h to allow the growth of enriched bacteria.

### 2.3. Isolation and Screening for Cellulolytic Bacteria 

After incubation of the BMS-CMC agar plates at 37 °C, the bacterial colonies were picked and streaked individually on LB agar. The fully grown bacterial isolates were then purified through a repeated streak plate technique followed by screening for CMC degradation. The cellulase activities of the isolated bacteria were semi-quantitatively tested by spot inoculation of the individual colonies on BMS-CMC agar plates. The inoculated plates were incubated for the growth of cellulolytic bacteria at 37 °C for 24–48 h. After the proper growth and incubation, the plates were stained with Gram’s iodine staining solution to observe the zone of CMC clearance around the bacterial colonies. The cellulase activity was estimated in terms of hydrolytic capacity (HC) which is defined as the ratio of the diameter of CMC clearance (zone) to the diameter of the bacterial colony. Subsequently, the cellulase-positive bacteria were identified by using the matrix-assisted laser desorption ionization–time of flight (MALDI-TOF) technique and characterized for further analyses.

### 2.4. Biotyping by MALDI-TOF

Initially, the isolated and purified bacteria were identified by the MALDI-TOF method. For biotyping, single colonies of each bacterium were seeded separately into 50 mL tubes containing freshly prepared LB broth and grown overnight at 37 °C by shaking at 150 rpm. After proper growth, the cultures were centrifuged for 5 min at 5000 rpm to harvest the bacteria. The samples were prepared by using the method of Schulthess and coworkers [33] with a few modifications. Briefly, the obtained bacterial cells were treated with 600 μL distilled water and 1.8 mL ethanol followed by repeated centrifugation at 10,000 rpm for 2 min to remove the residual ethanol. The pellets were air-dried thoroughly and re-suspended in 40 μL formic acid–water (70:30 *v*/*v*) solution followed by mixing with an equal volume of acetonitrile. The suspension was centrifuged at 10,000 rpm for 2 min, and 2 μL of each supernatant was transferred to the target plate. The bacteria on the target plate were overlayed with α-cyano-4-hydrocinnamic acid (CHCA) matrix solution (1 μL for each sample). Biotyper software was employed to compare the sample mass spectra with the mass spectra of reference strains in the MALDI database. The Biotyper software calculated the arbitrary unit scores above 1.85, indicating the similarity between the isolates and reference bacteria. The variations in MALDI-TOF scores were measured by fixing the species cutoff values at ≥1.9 while maintaining genus cutoff values at 1.8 followed by re-interpreting the top 2 matching database records in a Bruker Biotyper system (Bruker, Billerica, MA, USA). 

### 2.5. Identification and Phylogenetic Analysis

For molecular identification, the genomic DNA (gDNA) was extracted from the freshly cultured bacteria by using a soil DNA extraction kit (HiMedia Pvt Ltd., Mumbai, India), and quantified by using a NanoDrop spectrophotometer. The 16S rRNA gene was PCR amplified by using the bacteria-specific primer pair 27F (5′-AGAGTTTGATYMTGGCTCAG-3′) and 1492R (5′-TACGGYTACCTTGTTACGACTT-3′) [34]. The 50 μL PCR reaction contained 2 µL (10 ng/mL) of template DNA, 5 µL of *Taq* buffer (Bangalore Genei, India), 0.2 mmol/L of dNTPs (Bangalore Genei, India), 10 pmol of each primer, and 0.5 U of *Taq* polymerase (Bangalore Genei, India). The thermal cycling conditions consisted of initial denaturation at 95 °C for 5 min, 35 amplification cycles of primary denaturation at 94 °C for 1 min, annealing at 55 °C for 1 min, and extension at 72 °C for 1 min, followed by final extension of 7 min at 72 °C. The PCR products were checked for size and purity on 1.2% (*w*/*v*) agarose gels, and then sequenced by using the ABI Big-Dye version 3.1 sequencing kit as per the manufacturer’s instructions. The obtained 16S rDNA sequences were edited manually using ChromasPro software (http://www.technelysium.com.au/ChromasPro.html, accessed on 12 March 2021) and then compared with closely related sequences in the NCBI database (http://www.ncbi.nlm.nih.gov, accessed on 18 February 2021) using the BLASTn program. The phylogenetic tree was reconstructed in the MEGA X program [35] by maintaining a bootstrap value of 100. The phylogenetic tree was annotated in FigTree software v1.4.3. The 16S rDNA gene sequences of the reference strain and isolated bacterium-encoded RSP75 were aligned in the EMBOSS: dotmatcher program [36] using a window size of 30 and threshold value 40.

### 2.6. Biochemical Characterization 

The morphological and cultural characteristics, such as colony appearance, cell shape, cell motility, etc., of the potential strain were determined by microscopy. Other tests, such as catalase, Methyl Red (MR) and Voges–Proskauer (VP) reactions, indole production, citrate utilization, and hydrolysis of sugars or nitrogenous compounds, were carried out by using the HiBacillus test kit. The HiBacillus^TM^ identification kit is a standardized micromethod that combines 12 conventional and assimilation test sets to identify purified bacteria (HiMedia Pvt. Ltd.). The inoculated strips of the kit system were incubated for 18 to 24 h at 37 °C then they were read by referring to the standardized interpretation tables.

### 2.7. Effect of pH and Temperature on Cellulase Activity

To check the effect of pH on bacterial growth and cellulolytic activity, the isolate was grown at 37 °C for 24–48 h on BMS-CMC agar media with different pH in the range of 3.0–9.0. For optimization of temperature, the test isolate was grown for 24 h on the medium with optimum pH at different temperatures ranging from 30–50 °C. After incubation, the plates were stained with Gram’s iodine solution to check the zone of CMC clearance around the colony. The CMC clearance zones were measured by using the procedure as described above. 

### 2.8. Growth Curve 

To assess the growth pattern of the potential bacterium, a single colony was inoculated in BMS broth containing 0.5% CMC as the sole source of carbon and agitated at 150 rpm and 37 °C. The optical density (OD) was documented every 8 h using a side-arm flask till the bacteria achieved a declining phase. The OD and transmittance of the samples were recorded at λ600 on a colorimeter.

### 2.9. Enzyme Assays

To determine the production of lignocellulolytic enzymes, the BMS broth containing 0.5% (*w*/*v*) of different substrates, such as CCP, SD, GS, WH, FP, and SCB, in individual flasks were inoculated with 1% of freshly prepared bacterial cultures and incubated for 14 days by agitating at 150 rpm, 37 °C. The sample aliquots collected after different time intervals were centrifuged at 12,000 rpm for 10 min and 4 °C. The supernatants obtained were treated as crude enzyme extracts. The endoglucanase and xylanase assays were estimated as described previously [30]. The β-glucosidase assay was carried out as per the method of Gao and colleagues [37] with minor changes. Briefly, 1 mL of 0.2% cellobiose in sodium citrate buffer was treated with 1 mL of enzyme extract. After incubation for 60 min at 40 °C, the reaction was terminated by adding 3 mL of 3, 5-dinitrosalicylic acid reagent (DNSA) and boiling for 5 min in a water bath. The glucoamylase activity was assayed by mixing 250 μL of the substrate (1% starch in PBS, pH 7.4) with 250 μL of the enzyme. The reactions were carried out at 40 °C for 10 min and then terminated with 750 μL of DNSA reagent [38]. Thereafter, the reaction mixtures were boiled for 5 min in a water bath to denature the enzyme and allow color change. Finally, the absorbances were recorded spectrophotometrically at λ540 and the reducing sugars were estimated by using glucose as standard for endoglucanase and β-glucosidase assays. However, xylose was used as standard sugar for xylanase estimation. The protein concentration in the enzyme extracts was determined by Lowry’s method [39] using bovine serum albumin (BSA) as standard. The enzyme activities were calculated in international units (IU) where 1 IU of activity is defined as the amount of enzyme required to liberate 1 μMol of glucose equivalents per min under the standard assay conditions.

### 2.10. Determination of Substrate Degradation Ratio

The effect of bacterial treatment on the degree of substrate degradation was characterized by seeding 1 mL of freshly prepared inoculum (OD600:0.5) in BMS media containing 1000 mg of individual substrates as carbon sources. The culture media were incubated on a rotary shaker at 37 °C and 150 rpm. During the incubation period, the culture flasks were continuously checked for a change in turbidity of the media and bacterial growth as a measure of degradation of the substrates. The inoculated culture media containing different substrates, viz., CCP, FP, GS, SCB, SD, and WH, were incubated for up to 8 days. The substrate degradation ratio was calculated using the method described by Du and coworkers [40] with a few modifications. The culture broth after proper incubation was centrifuged at 12,000 rpm for 15 min to recover the unused substrates as biomass. The pellet containing biomass with bacterial cells was treated with acetic–nitric acid reagent (1 M) followed by a wash of distilled water. Subsequently, the biomass was washed with absolute ethanol for 20 min for complete removal of the bacterial debris. The unused substrates were then air-dried and finally weighed to measure the residual LC. The respective controls contained individual substrates but no bacterial treatments. The percent (%) substrate degradation was defined as the ratio of the final weight of substrates to the initial weight of substrates.

### 2.11. Electron Microscopy and Characterization of Hydrolyzed Substrate

The characterization and comparison of the treated and untreated substrates were carried out by various techniques such as field emission scanning electron microscopy (FESEM) and Fourier transform infrared spectroscopy (FTIR). The hydrolysis of the FP was analyzed as described previously [41]. For these analyses, the freshly prepared BMS media containing individual substrates, such as CCP, FP, SCB, WH, etc., were seeded with 1% of the bacterial inoculum, and incubated at 37 °C, 150 rpm. After incubation, the broth was centrifuged at 5000 rpm for 5 min to harvest the biomass containing bacterial cells. For FESEM analysis, the samples were prepared by using the method of Galabova et al. [42]. The specimens were adequately air-dried and coated with 100 Å gold and then observed at a voltage of 10 kV in a Nova Nano SEMNPEP 303 (FEI Technologies, Oregon, USA).

For functional group analysis, the FTIR spectroscopy was carried out for FP that was used as the sole source of carbon for bacterial growth. The FP discs (10 mg) obtained from bacterial treatment and control experiments were individually mixed with 1000 mg of spectroscopy grade potassium bromide (KBr) in an agate mortar and subsequently pressed into discs. The infrared (IR) spectra were examined on an FTIR spectrometer (Jasco 6100, Germany). The FTIR spectra of the hydrolyzed and untreated FP were recorded in the absorption band mode ranging from 400 to 4000 cm^−1^ with a resolution of 4 cm^−1^ accumulating 32 scans. 

### 2.12. GC-MS Analysis

To determine the byproducts of saccharification and fermentation, freshly prepared BMS medium containing WH as substrate was inoculated with freshly grown potential bacteria, and incubated at 150 rpm for 8 days to allow the accumulation of reducing sugars. Based on the higher enzyme activities and degradation of the tested agro-wastes as substrates, WH was selected as a sole source of carbon for the saccharification purpose. After the incubation period, the medium-containing flasks were seeded with 0.1%, of *Saccharomyces cerevisiae* for fermentation of the reducing sugars. The media were incubated at 30 °C for 48 h followed by centrifugation at 10,000 rpm for 10 min to obtain the supernatant. The acquired supernatants were subjected to filtration through a 0.2 μm syringe filter. Finally, the ethanol formed was estimated by GC-MS analysis using the method previously described by Bagewadi and colleagues [43]. The identity of the mass spectroscopic peaks was confirmed from the database of the National Institute of Standards and Technology (NIST). However, the survey was restricted to mass numbers 15, 30, 42, and 45 where fermentation products yield the majority of ions.

### 2.13. Statistical Analyses

The data were analyzed statistically by one-way analysis of variance (ANOVA) using Tukey’s test to determine the degree of significance in SPSS software version 22 (IBM SPSS, New York, NY, USA). A *p* value < 0.01 is considered significant. The obtained results are demonstrated as mean ± standard deviation of three independent replicates unless stated otherwise. The graphs were plotted in Origin software version 8.1 (Origin Lab. Corporation, Northampton, MA, USA).

## 3. Results

### 3.1. Identification of the Insect

**Figure 1:**
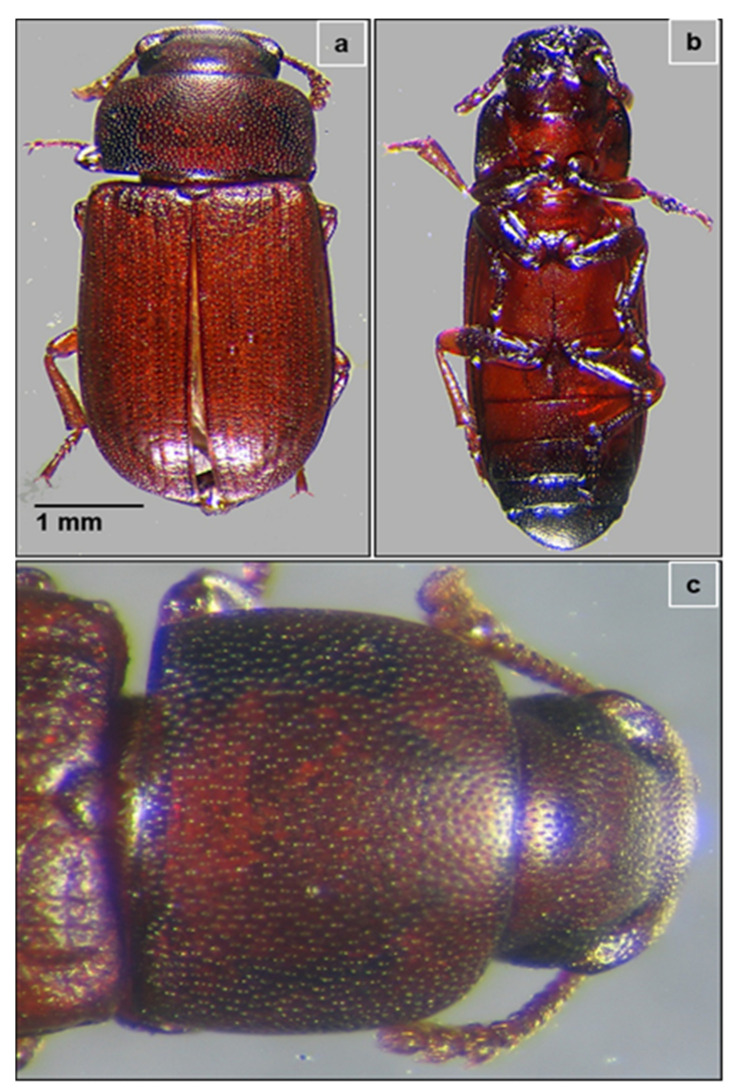
The red flour beetle, *Tribolium castaneum.* (**a**) Dorsal view showing the flattish curved-sided body covered with minute punctures. Ventral view of the whole body of adult beetle (**b**), and magnified view of the thorax with prominently large eyes and capitate antennae with broad segments (**c**).

In the present study, the collected insects were identified by using dichotomous keys based on morphological characteristics and then confirmed by the Zoological Survey of India, Pune, India. The “armor-like” elytra hiding the membranous hind wings showed that the collected insect was a beetle species. Further, reddish-brown color, approximately 3 mm long, with a flattish body and curved thorax (Figure 1a–c) confirmed it as a red flour beetle, *Tribolium castaneum*. The head and upper part of the thorax were covered with minute punctures and the wing cases (elytra) were ridged along their length. Capitate antennae were enlarged with the last three segments prominently wider than the proximal segments. Moreover, some of the beetles were observed to show short flights during experimentation.

### 3.2. Isolation and Identification of Bacteria by MALDI-TOF

When the gut extracts were spread on BMS-CMC and LB agar media, many bacterial isolates grew on LB agar plates. However, after the repeated streaking on BMS-CMC agar media, 15 isolates were sustained and grew, indicating their potential to utilize CMC. Since MALDI-TOF-based identification is faster and more accurate than traditional biochemical characterization, it was employed to identify the bacteria isolated from the beetle gut. The Biotyper software-based comparison resulted in an arbitrary unit score ranging from 1.802 to 2.244, reflecting the similarity of the isolated bacterial species to reference spectra of best matches from the database. The MALDI-TOF analyses affiliated isolates to genera including *Bacillus*, *Citrobacter*, *Kluyvera*, *Escherichia*, *Enterococcus,* and *Achromobacter*, considering mass spectroscopic scores of over 1.8 (Table 1). Based on MALDI-TOF analysis, the isolate encoded RSP75 was identified as *Bacillus* species with a unit score of 2.207.

### 3.3. Screening and Selection of the Potential Bacteria 

Of the 15 bacterial cultures that were isolated and grown on BMS-CMC agar media, 12 isolates showed varying cellulolytic activity evident from zones of CMC clearance in the range of 4 to 28 mm in diameter (Figure S1A). Among these, 46.66% bacteria showed lower activity from 4–10 mm whereas 26.66% showed medium activity (11 to 20 mm). Noteworthily, the isolate RSP75 showed a larger zone of CMC clearance measuring 28 mm in diameter. As 20% of the isolates did not show any activity, they were eliminated from further study. The hydrolytic capacity ratio of the cellulase-positive isolates ranged from 1.5 to 5.5 (Figure S1B). Since RSP75 showed maximum cellulolytic potential, it was selected for further analysis and identified by a molecular phylogenetic approach.

### 3.4. Molecular Identification and Phylogenetic Analysis of the Bacteria

**Figure 2:**
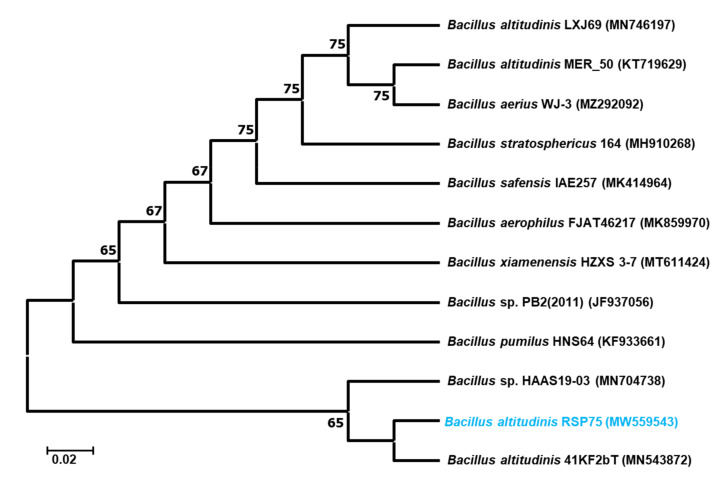
The phylogenetic reconstruction of *B. altitudinis* RSP75 generated by the neighbor-joining method in MEGA X, showing its relationships with closely related bacterial species from NCBI. Accession numbers are given in parentheses, and the bootstrap values are shown on nodes when above 60. Scale bar represents 0.02 Jukes–Cantor distances.

The selected isolate with a potential cellulose-degrading repertoire was identified by 16S rRNA gene sequencing and amplification. The BLASTn analysis and phylogenetic reconstruction of the RSP75 showed over 99.46% sequence similarity to *Bacillus altitudinis*. The 16S rRNA sequence similarity of RSP75 was also confirmed by EZbiocloud (https://www.ezbiocloud.net/identify, accessed on 5 July 2021) where it showed 99.23% similarity to *B. altitudinis* 41KF2b(T). Further, upon the phylogenetic reconstruction, RSP75 formed a cluster with *B. altitudinis*, therefore, it is hereafter referred as *B. altitudinis* RSP75 (Figure 2). The two-dimensional matrix plot showed a slight frameshift between the 16S rRNA gene sequences of the reference strain and *B. altitudinis* RSP75 (Figure S2). This slight frameshift could have resulted from mutations due to differences in habitat and corresponding metabolic shifts of the bacteria. The 16S rRNA gene sequence of *B. altitudinis* RSP75 can be accessed from the NCBI database under accession number MW559543.

### 3.5. Biochemical and Growth Curve Analyses

**Figure 3:**
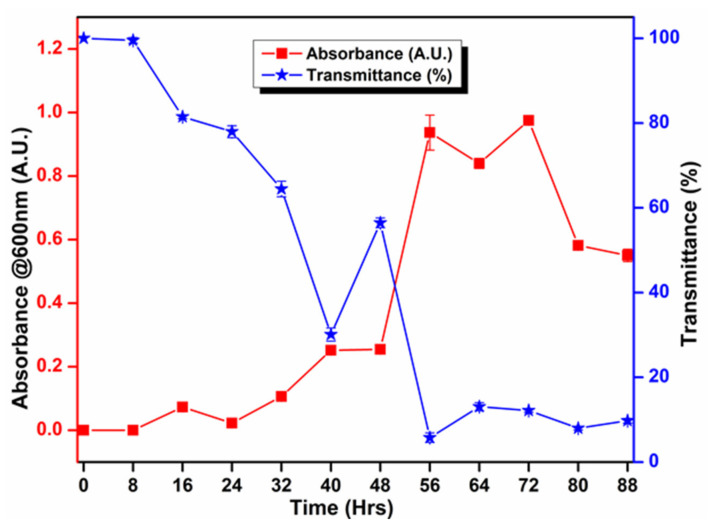
Growth pattern of *B. altitudinis* RSP75 in BMS medium supplemented with CMC as the sole source of carbon.

The principle of biological identification is based on the ability of bacteria to metabolize particular types of chemical compounds. The biochemical analysis indicated that *B. altitudinis* RSP75 utilizes a variety of carbon and nitrogen sources, such as ornithine, phenylalanine, sucrose, glucose, mannose, and cellobiose. In addition, the bacterium was also found positive in the Voges–Proskauer test. Subsequently, *B. altitudinis* RSP75 also showed enzyme activities, namely β-glucosidase, pyrrolidonylarylamidase (PYR), and hydrolysis of *p*-nitrophenyl β-d-glucopyranoside. However, it showed negative reactions towards substrates such as lysine, urease, indole, sorbitol, trehalose, melibiose, salicin, raffinose, lactose, etc. Similarly, it also lacked α-galactosidase and β-xylosidase activities. *Bacillus altitudinis* RSP75 showed oxidation of glucose in the Methyl Red test. Moreover, *B. altitudinis* RSP75 initially exhibited very slow growth on BMS-CMC media up to 16 h, which was followed by continuous increments from 16 to 72 h (Figure 3). The growth curve profile further displayed a declining trend in cell growth after 80 h. 

### 3.6. Optimization of pH and Temperature for the Bacterium

**Figure 4:**
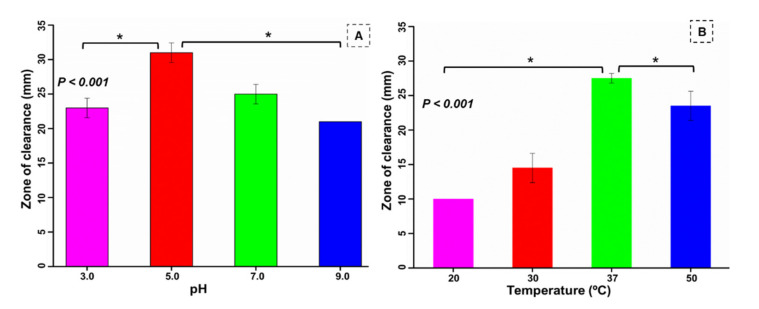
Effect of pH (**A**) and temperature (**B**) on the cellulose-degrading activity of the potential bacterium. Results are presented as average ± standard deviation of five experimental replicates. The asterisk (*) over the bars indicates *p* value statistically significant at <0.01 for optimum pH (pH 5.0) and temperature (37 °C) when compared with other related conditions.

The physicochemical parameters, such as pH and temperature, of culture media are well known to influence the metabolism and catalytic efficiency of bacteria. The gut bacterium *B. altitudinis* RSP75 isolated in the present study showed good cellulolytic activities at pH 5.0 (*p* < 0.001), indicating it as the most suitable pH for cellulose degradation (Figure 4A). Thus, *B. altitudinis* RSP75 showed more efficiency at acidic pH than in alkaline conditions. Similarly, study of the effect of temperature demonstrated maximum activity (27.5 ± 0.7 mm) at 37 °C, indicating the mesophilic nature of *B. altitudinis* RSP75 (*p* < 0.001). Although the activity at 50 °C was 1.6-fold higher than at 30 °C, it was much lower than the activity observed at 37 °C (Figure 4B).

### 3.7. Enzyme Assays

**Figure 5:**
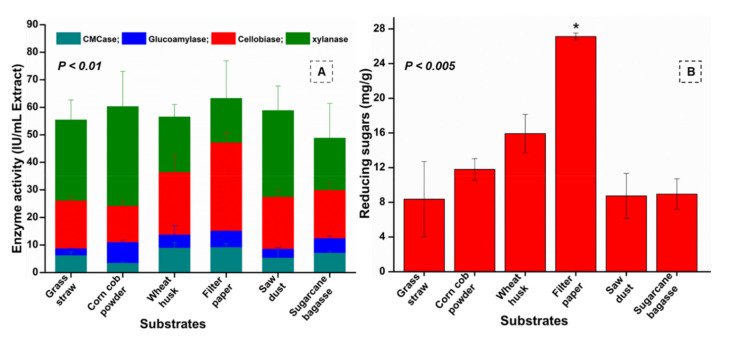
(**A**) Profile of the enzyme activities of *B. altitudinis* RSP75 on different carbon sources used as substrates after 8 days of incubation. (**B**) Reducing sugar production by *B. altitudinis* RSP75 on different substrates. The asterisk (*) indicates reducing sugar production statistically significant at *p* < 0.005 on FP when compared with other substrates.

Among the tested substrates, higher activities were achieved on substrates such as FP, WH, SD, and GS as compared to CCP and SCB. The highest endoglucanase activity of 9.08 ± 1 IU/mL extract, also known as CMCase activity, was exhibited by *B. altitudinis* RSP75 on FP (Figure S3a) followed by WH (8.89 ± 1.9 IU/mL extract) as substrates after 8 days of incubation. However, the lowest endoglucanase activity of 2.34 IU/mL extract was found with SCB as substrate on the first day of incubation. Similar to the endoglucanase assay, the highest β-glucosidase activities (47.1 ± 3.7 and 36.3 ± 6.3 IU/mL extract) were produced when *B. altitudinis* RSP75 was grown continuously for up to 8 days on FP and WH, respectively. In the case of β-glucosidase, the lowest activity (1.81 IU/mL extract) was achieved with SCB as the sole source of carbon on the 4th day of incubation. Among the tested agro-wastes used as substrates, the highest β-glucosidase activities were produced on WH, followed by SD and CCP. For xylanase activity, a unique trend of substrate preference was observed as a higher enzyme production of 63.2 ± 13 IU/mL extract was noted after 8 days of incubation on FP followed by CCP (60.2 IU/mL extract), indicating them as favorable substrates among the tested agro-wastes. On WH as a carbon source, the highest xylanase activity of 58.81 IU/mL extract (though lower than the activity on FP) was achieved after 4 days of incubation. However, the lowest xylanase activity of 7.78 ± 1.7 IU/mL extract was determined on day 1, when *B. altitudinis* RSP75 grew on SCB as substrate. In the case of glucoamylase activity, the highest activity of 15.05 ± 0.1 IU/mL extract was exhibited after 8 days on FP used as a sole source of carbon. After FP, the glucoamylase activity followed a trend of WH > SCB > CCP with 13.6 ± 3, 12.3 ± 0.9, and 10.9 ± 0.8 IU/mL of extracts, respectively. The maximum glucoamylase activity on GS was observed after 14 days of incubation, demonstrating a value of 9.42 IU/mL extract. Among the tested substrates, glucoamylase activity was lower using GS and SD as carbon sources. During the period of incubation, the enzyme activities were better on the 8th day, therefore this was taken into consideration for further analysis. Among the tested agro-wastes used as substrates, the overall enzyme activities were higher on WH. The enzymatic profile signified that the potential bacterium showed higher potential (eight-fold) for xylanase activity when compared with CMCase (*p* < 0.001). After xylanase, the potential bacterium showed maximum activities towards β-glucosidase, as shown in Figure 5A. Furthermore, the sugar production was highest in the case of FP and then WH, indicating them as potential carbon sources for fermentation (Figure 5B).

### 3.8. Determination of Substrate Degradation Ratio

**Figure 6:**
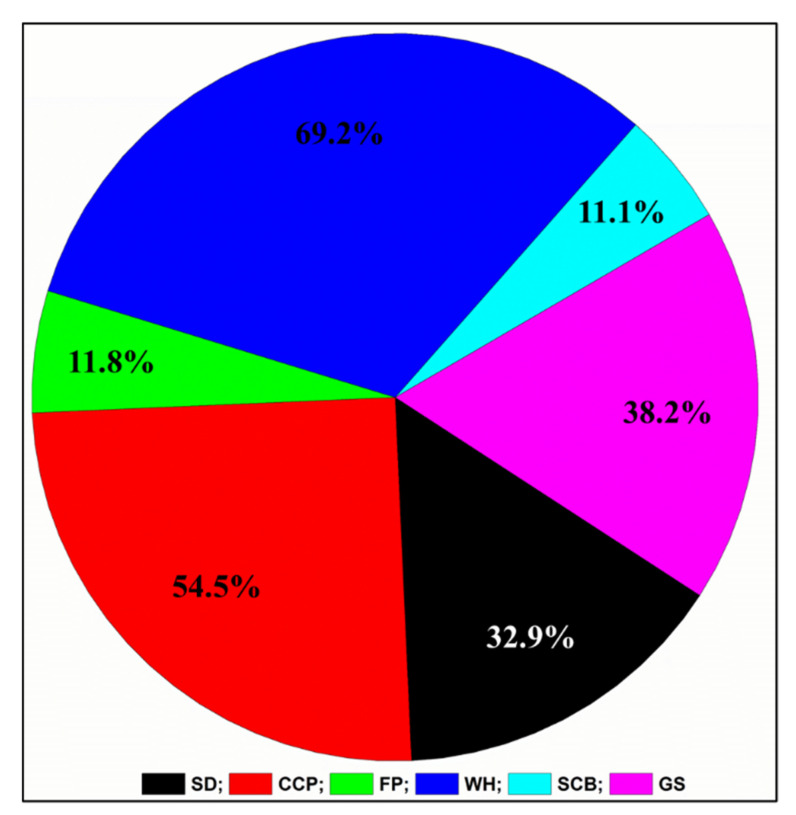
Percent (%) substrate degradation achieved with various agro-wastes by *B. altitudinis* RSP75. SD: sawdust; CCP: corn cob powder; FP: filter paper; WH: wheat husk; SCB: sugarcane bagasse; GS: grass straw.

Among the substrates, *B. altitudinis* RSP75 demonstrated the highest degradation of WH (69%) followed by CCP (54%; Figure 6). Being recalcitrant and purely cellulosic, FP was degraded the least (11%) among the tested carbon sources. The degradation of the substrates showed correlation with substrate compositions, as WH, and CCP are predominantly composed of many five- and six-carbon sugars such as mannose and other hemicelluloses. The biochemical analysis and enzyme assays demonstrated that they were efficiently degraded by *B. altitudinis* RSP75.

### 3.9. Scanning Electron Microscopy and Characterization of the Hydrolyzed Substrates

**Figure 7:**
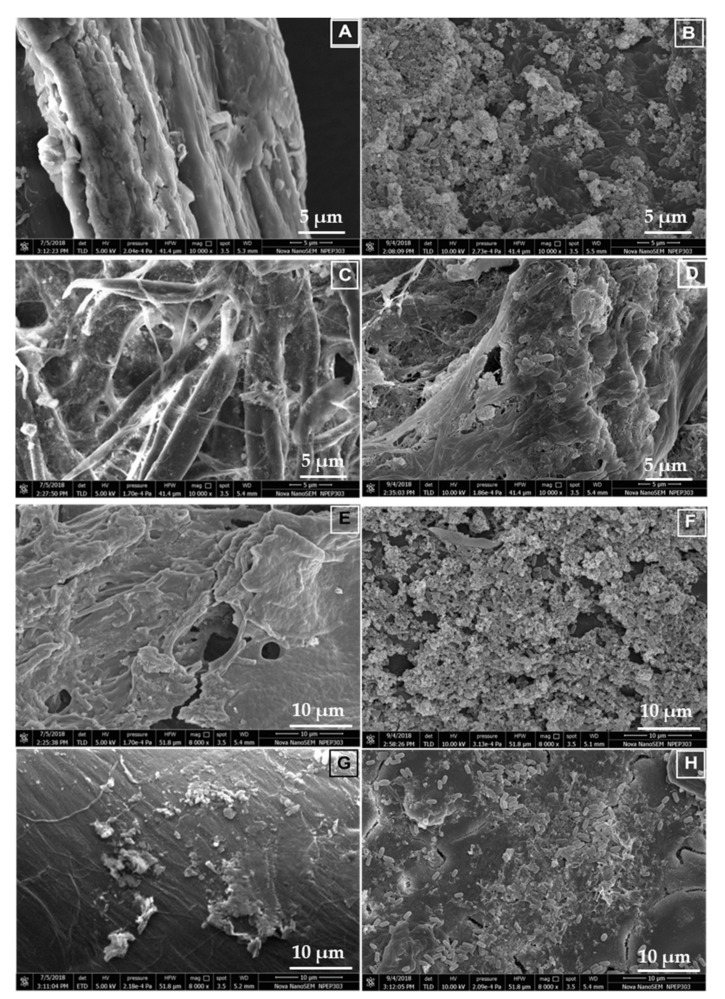
Hydrolysis of agro-wastes and FP used as substrates for the production of cellulases by *B. altitudinis* RSP75 after 8 days of incubation in BMS media. Field emission scanning electron micrograph of untreated corncob powder (**A**), treated corncob powder (**B**), untreated filter paper (**C**), treated filter paper (**D**), untreated sugarcane bagasse (**E**), treated sugarcane bagasse (**F**), untreated (**G**) and treated wheat husk (**H**).

The characterization of hydrolyzed substrates is imperative for a comprehensive understanding of the substrate degradation by bacteria. To this end, the effects of *B. altitudinis* RSP75 on agro-wastes and FP as substrates were determined by FESEM and FTIR techniques. The FESEM-based visualization and comparison of the surface topographies between control and treated agro-wastes showed that surface morphologies were marked by discernible structural changes (Figure 7). The control substrates had a smooth texture while the surfaces of treated agro-wastes were rough and porous. In the case of the tested substrates, the bacterial cells were found to make tunnels and pores, exposing the inner fibers of the cellulosic biomass, while in WH, *B. altitudinis* RSP75 catalyzed superficial hydrolysis of the biomass. Moreover, *B. altitudinis* RSP75 released microfibers from the long chains of cellulose, as can be seen in the micrographs of all tested substrates (Figure 7B,D,F,H). The disruption of LC biomass was also evident as high volumes of hydrolyzed biomass were found to accumulate on the surfaces of treated substrates. The rupturing of the substrates and adhesion of the bacterial cells further demonstrated the utilization of the agro-wastes as carbon or energy sources by the bacterium.

**Figure 8:**
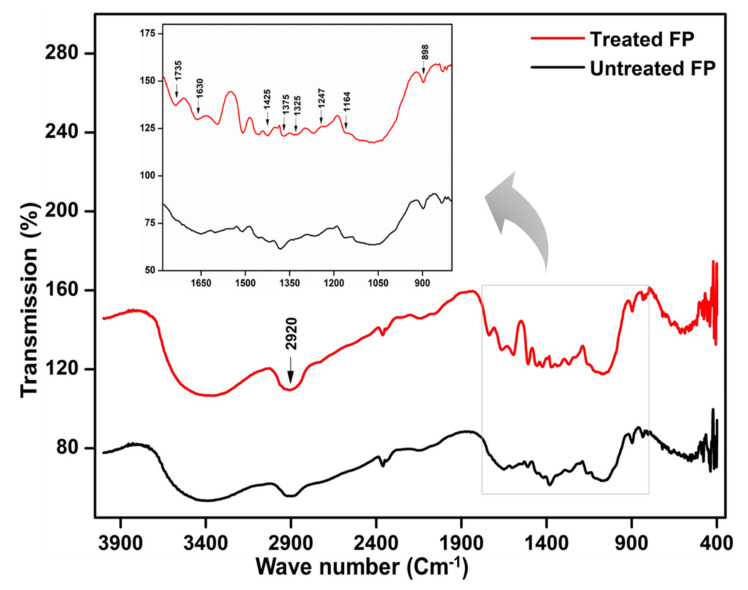
FTIR analyses of filter paper before and after hydrolysis by the bacterium *B. altitudinis* RSP75. The FP was used as the sole source of carbon for the growth of the bacterium.

The FTIR analysis showed subtle alteration of the functional groups due to enzymatic hydrolysis. The FTIR spectral profiles of untreated and treated FP showed marked differences in the areas that represent cellulose (Figure 8). A band at 2920 cm^−1^ is a characteristic of the methyl group (OCH_3_) present in cellulose. The intense absorption bands from 890 to 1750 cm^−1^ could be assigned to crystalline and amorphous cellulose contents of the FP. The IR band at 1164 cm^−1^ attributed to C-O-C represents an asymmetric stretching. The noteworthy difference between untreated and treated FP was a change in the intensity of the absorption band at 1735 cm^−1^. The hydrolysis of the FP was further supported by the decrease in the intensity of bands at 1247 and 1425 cm^−1^ which represent the stretching of the C-O bonds in cellulose. The reduction in the absorption bands at 1325 and 1375 cm^−1^ also indicated the degradation of the crystalline cellulose caused by *B. altitudinis* RSP75. The deformation of C-H and stretching of the C=O bonds at 1425 and 1630 cm^−1^, respectively, elucidate the reduction in band intensities, depicting cellulose hydrolysis. Lastly, the intensification of the bands at 898 cm^−1^ is indicative of the degradation of amorphous contents in cellulosic materials. All these structural alterations infer the utilization of FP as a carbon source for metabolite (reducing sugar) production by the bacterium. 

### 3.10. GS-MS Analysis

To propose the possible mechanism of cellulose bioconversion by the gut bacterium *B. altitudinis* RSP75, the metabolic products were detected and identified by mass spectrometry. The GS-MS analysis identified the compounds released during hydrolysis and fermentation of LC wastes. The total ion chromatographic (TIC) profile of the compounds derived from hydrolyzed biomass in fermentation culture is shown in Figure S4, whereas the identities of the obtained peaks are presented in Table 2. The TIC profile of the hydrolysate displayed a large number of peaks, indicating the release of many low-molecular-weight compounds from the agro-waste due to cellulolytic activities of *B. altitudinis* RSP75. The low-molecular-weight compounds, such as ethanoic acid, methane carboxylic acid, acetasol, methyl ethanoate, acetone alcohol, methyl ester, and pyruvic acid, were detected in the culture broth. The most important peaks elucidating the fermentation were assigned to carbon dioxide and acetate, showing a retention time (RT) of 1.490 and 2.025, respectively. Ethanol is usually known to show a peak from *m*/*z* = 41 to 45 which is quite clear in the mass spectrum of the hydrolyzed broth due to co-culturing of *B. altitudinis* RSP75 with *S. cerevisiae*. The mass peak at 29 *m*/*z* represented the presence of the acetaldehyde compound. The peaks corresponding to 2-propanone (acetol) and propanoic acid (acetic anhydride) showed RTs of 2.275 and 3.115, respectively. Carbon dioxide is known to contribute most ions to mass number *m*/*z* = 44 which was also observed in the present study. Being the intermediate metabolites of cellulose fermentation, propionate and methyl pyruvate signpost the breakdown of cellulose by the bacterium. Based on our investigation and the FTIR and GC-MS analyses, together with enzyme assays, a schematic pathway was proposed for the hydrolysis mechanism of *B. altitudinis* RSP75 which is elucidated in the discussion section.

## 4. Discussion

The process of bioprospection leads to the discovery of promising and potential microbes for industrial-scale biomass conversion. In this context, the physiologically diverse gut systems of insects represent unique niches to prospect for bacteria that can be harvested for bioengineering and consolidated bioprocessing. In recent years, insect gut systems have been proven as useful resources for enzymes with various industrial applications. Among insects, termites have been the primary focus for scientists to isolate LC-degrading gut bacteria [44]. Thus, the present study represents an additional group of insects that can be considered for bioprospection as they efficiently digest LC [45]. Despite being a well-known model system for multidimensional research in food safety and molecular sciences [46], currently little is known about the LC-degrading bacteria in the gut system of the tenebrionid beetle *T. castaneum*. To this end, the present study explored the cellulose-degrading bacteria from the gut system of *T. castaneum* and suggests it as a potential reservoir for lignocellulase-encoding proteins. Being a reliable, rapid, easy, and economic technique, MALDI-TOF mass spectrometry is commonly applied for the identification of Gram-positive bacteria [47]. To date, a number of cellulolytic bacteria have been identified using MALDI-TOF analyses [48,49]. In the present study, MALDI-TOF analysis indicated the presence of *Bacillus*, *Citrobacter*, *Kluyvera*, *Escherichia*, *Enterococcus*, *Achromobacter*, etc. in the gut of the beetle. Many researchers have described that *Enterococcus*, *Escherichia*, *Kluyvera,* and *Citrobacter* spp. are involved in a variety of functions in insects [50,51]. It has been established that these gut microbes play a significant part in the defense as well as nutrition of the host insects [52]. In particular, the majority of the Firmicutes that inhabit the gut systems of insects are mainly involved in carbohydrate depolymerization and nitrogen metabolism [51,53,54]. Likewise, the gut bacteria of *T. castaneum* might help the host in the digestion of wheat bran which is predominantly rich in dietary fibers such as xylans, cellulose, lignin, galactan, and fructans [18].

To characterize the CMCase activity of the isolated strains, different experimental assays were performed by using plate-based as well as analytical methods. The results of these experiments revealed that not all bacteria can grow and degrade CMC, however, a few strains of *Citrobacter* species and *B. altitudinis* RSP7 showed conspicuous CMCase activities. However, low, or the absence of, cellulolytic activity in some isolates suggested their primary functionalities other than carbohydrate digestion within the insect gut [55]. Of the 15 bacteria isolated in the present study, *B. altitudinis* RSP75 showed a relatively higher potential to degrade cellulose which was also evident from the downstream testing assays. The cellulose hydrolytic ratio and zone of CMC clearance by *B. altitudinis* RSP75 was much larger than the clearance zone by bacteria previously isolated from the larvae of *Dendroctonus armandi* [56]. 

To date, members of the genus *Bacillus* have been identified in many species of insects, such as scarab beetles, such as *Holotrichia parallela* [57], *D. armandi* [56], moths like *Diatrea sacharalis* [55], termite, *C. formosanus* [23], and snails [29,30,58], which are mostly cellulose-digesting animals [59,60,61,62]. However, to the best of our knowledge, isolation of *B. altitudinis* from an insect has not been reported previously, although its presence has been established in fish gut [63]. In this regard, the present investigation is the first study to report the presence of *B. altitudinis* strains in insect gut systems. Furthermore, the cellulolytic repertoire of *B. altitudinis* has not been characterized on agricultural wastes despite a few reports that focused on process optimization using simulations [63] but different isolation sources (Table S1). *Bacillus altitudinis* RSP75 is a spore-forming, white-colored, rod-shaped, Gram-positive, aerobic, and mesophilic bacterium. The biochemical characteristics of *B. altitudinis* RSP75 were in line with earlier reports [64,65]. From the biochemical characterization, it is evident that *B. altitudinis* RSP75 can utilize many polysaccharide components of plant cell walls such as cellulose, xylan, starch, and mannose. Our observations are in congruence with those of Vettath and coworkers who stated that many genes (875 out of 3820 protein-coding genes) in *B. altitudinis* encode for carbohydrate and protein metabolism [66]. Since mannan constitutes an important component of prebiotics for animal husbandry and nutritional supplements [67], *B. altitudinis* RSP75 could prove to be a promising candidate for mannan oligosaccharide-based dietary supplements of dairy and feedstock. *Bacillus altitudinis* RSP75 also showed a growth pattern similar to *B. megaterium* S3, showing slow and extended growth on CMC media [68] due to a metabolic shift to use cellulose as a carbon source. One of the peculiarities of *B. altitudinis* RSP75 is the ability to grow under acidic pH because relatively few bacterial species are known to tolerate acidic conditions, particularly in nature [69]. The acidophilic nature of *B. altitudinis* RSP75 signifies that its enzymes are likely to function well at acidic pHs prevailing in biomass conversion processes. The acidophilic enzymes have a variety of applications such as in feedstock, baking, and biofuel industries [70].

The enzymatic profile and substrate degradation revealed that *B. altitudinis* RSP75 can effectively degrade agro-residue-based substrates such as GS, WH, and SCB, showing significant production of cellulolytic and hemicellulolytic enzymes responsible for LC degradation. This, in other words, signifies that *B. altitudinis* RSP75 could be employed to valorize the waste sector into a green useful materials sector for the production of high-value biochemicals. The significant observation of the present study is the secretion of complex LC-hydrolyzing enzymes such as xylanase, β-glucosidases, endoglucanase, and glucoamylase. The good xylanase, β-glucosidases, and glucoamylase activities observed in *B. altitudinis* RSP75 demonstrate positive corroboration of the xylan- and starch-rich diet of the beetle (host). Since starch comprises 16% of the dry weight of wheat bran [71], *B. altitudinis* RSP75 may prove very useful for its waste disposal management and subsequent bioconversion. However, β-glucosidase activities of *B. altitudinis* RSP75 were lower than the activity of *B. altitudinis* YC-9 (Table 3). The observed difference could have been due to the different assay conditions used. In addition, spring silt as a different source of isolation for *B. altitudinis* YC-9 could have contributed to the observed differences in enzyme activity [65].

The topographical changes in tested substrates including FP and WH are important considerations that indicate disruption of the polymer due to bacteria, signifying the secretion of hydrolytic enzymes [83]. The FESEM imaging of the treated CCP, SCB, WH, and FP exhibited cracking and delamination of substrates. Similar observations were also made by many researchers in the case of wood composites treated with bacteria and fungi [84,85]. These observations also corroborated previous reports where authors stated cracking and loosening of rice straw due to bacterial treatment [86]. The authors further stated that microbes initially devour the outer polymers of the substrates, releasing toxic metabolites in the form of esterases or organic acids. The released compounds damage the surfaces of the polymer, hence exposing inner chains of the composite which are susceptible to enzymatic action, thereby accelerating degradation [87].

Due to structural complexity, the LC compounds are recalcitrant to enzymatic hydrolysis [88]. The substrate degradation potential (32–69.2%) of *B. altitudinis* RSP75 was much higher than the degradation capacity (10.7%) of many previously reported *Bacillus* [89] and *Klebsiella* species [12]. Among the tested agro-wastes, the maximum degradation was achieved with WH followed by CCP and GS. WH is a renewable and rich source of hemicellulose contents, including xylans, galactan, and fructans, along with some minerals [90]. Since it is abundantly available, its bioconversion could prove a vital and sustainable source of energy alongside feedstock for animals [91]. The substrate degradation ratio and FESEM results were in congruence with the FTIR spectral analysis. The prominent changes observed were the removal of the bands representing cellulose at 1630 and 1735 cm^−1^ corresponding to the C=O stretching of the aldehyde groups of the carbon source [92,93,94,95]. The FTIR observations were also in agreement with many earlier studies [96,97]. The absorption peaks at 1320 and 1425 cm^−1^ were assigned to the symmetric CH_2_ wagging and bending while the absorption band of ~1375 cm^−1^ represented bending of C-H bonds [98,99]. The absence of these bands in the treated substrate congruently indicated *B. altitudinis* RSP75-mediated hydrolysis.

**Figure 9:**
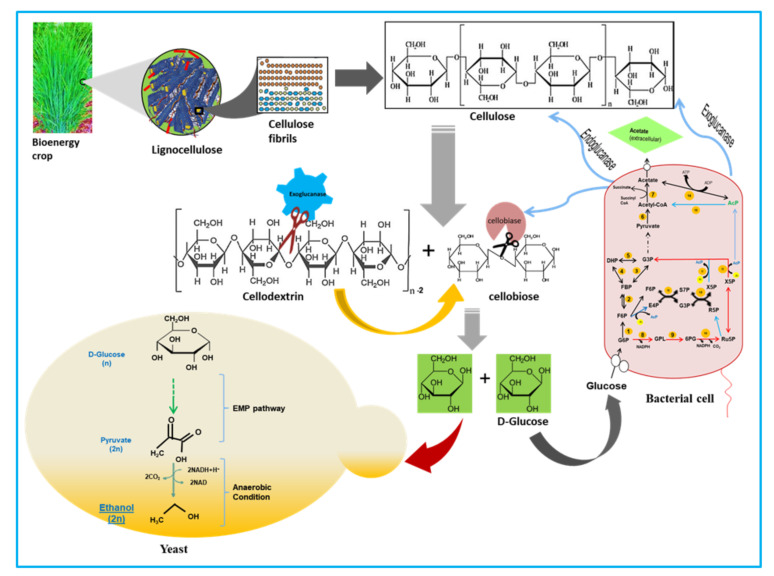
Predicted mechanism/pathways used by *B. altitudinis* RSP75 for the metabolism of cellulose with synergism with yeast strain for ethanol production. G6P: glucose-6-phosphate; GPL: 6-phospho glucono 1, 5 lactone; 6PG: 6-phosphogluconate; Ru5P: riulose-5-phosphate; X5p: xylulose-5-phosphate; R5P: ribose-5-phosphate; S7P: sedoheptulose-7-phosphate; G3P: glyceraldehyde-3-phosphate; F6P: fructose-6-phosphate; E4P: erythrose-4-phosphate; F6P: fructose-6-phosphate; FBP: fructose-1,6-bisphosphate; DHP: dihydroxyacetone phosphate; AcP: acetyl phosphate. The numbers in yellow circles indicate enzymes, 1: glucokinase; 2: phosphoglucokinase; 3 and 4: aldolase; 5: isomerase; 6: pyruvate dehydrogenase complex; 7: pyruvate oxidase; 8 and 9: glucose-6-phosphate dehydrogenase; 10: 6-phosphogluconate dehydrogenase; 11: bifunctional phosphor ketolase; 12: transaldolase; 13: transketolase; 14: acetate kinase; 15: phosphotransacetylase.

During co-culture, the bacterium, *B. altitudinis* RSP75 provided sugars to the yeast for which it primarily attacked the β-1,4 linkage of the cellulose fibrils, releasing either the short chains of cellulose or terminal disaccharide units, i.e., cellobiose (Figure 9). The released cellobiose units are hydrolyzed further by cellobiase into a six-carbon sugar, glucose. Similarly, hemicellulose, where xylan occupies the major part, is acted upon by different endoxylanases, releasing short stretches of xylan which are then broken down by exoxylanases into five-carbon sugars. These five- and six-carbon sugars are ingested by the yeast as well as bacterial cells and metabolized primarily in the Embden–Meyerhof–Parnas pathway. The two units of the pyruvate generated from each sugar molecule are then converted into acetaldehyde and subsequently oxidized into CO_2_ and ethanol in the mitochondria via anaerobic fermentation by the yeast. The majority of these chemical compounds were identified in the culture broth in terms of the mass spectra and *m*/*z* values (Table 2). The mass spectra of the metabolites revealed major peaks with retention times of 1.4, 2.05, 2.27, 3.11, 4.88, and 8.15 min. Moreover, the GS-MS analysis showed three characteristic peaks for ethanol, carbon dioxide, and acetaldehyde. The peak with a molecular mass of 45 and retention time of 2.523 is characteristic of ethanol [43]. The peaks at 29 and 44 signified the production of chemical compounds such as acetaldehyde and CO_2_, respectively [100]. To date, significant progress has been made for ethanol fermentation from LC by using co-cultivation of microorganisms. Recently, Harish and colleagues reported 0.41 g/g of ethanol produced from banana waste using co-culture of *C. thermocellum* and *C. thermosaccharolyticum*. The authors also mentioned that co-cultivation was more effective for cellulose hydrolysis and reducing sugar production and bioconversion [101]. Similar to previous reports, we observed that co-culturing of *B. altitudinis* RSP75 and yeast significantly improves the rate of LC bioconversion and ethanol yield within a short duration of fermentation [102,103].

All of the above characteristics demonstrate the potential lignocellulolytic repertoire of *B. altitudinis* RSP75, and therefore encourage its further characterization for LC bioconversion. The noteworthy contribution of our study is the isolation of the *B. altitudinis* RSP75 strain from the gut system of *T. castaneum* bearing significant lignocellulolytic potentials. Isolation of *B. altitudinis* RSP75 from *T. castaneum* and its subsequent characterization also suggests its supplementary role in the host in cellulose digestion. Whether *B. altitudinis* RSP75 represents a valuable metabolic collaborator with *T. castaneum*, enhancing its ability to dwell on cellulosic plant matter, warrants further consideration. Moreover, further studies are needed to elucidate if these bacteria are indeed more efficient in degrading cellulose by cloning individual cellulase-encoding genes. Nevertheless, the obtained results are encouraging and suggest further investigation of the efficiency and cellulolytic activity of cellulases and hemicellulases from the potential strain *B. altitudinis* RSP75 for possible application in bioconversion of WH and SCB into 2nd generation bioethanol or other processes.

## 5. Conclusions

The present study demonstrated the gut of *T. castaneum* to be a unique and potential resource for the bioprospection of efficient cellulose-degrading bacteria. The potential isolate identified as *B. altitudinis* RSP75 produced many lignocellulose-hydrolyzing enzymes that could be utilized to break down agro-wastes into readily usable commodity chemicals. The substrate degradation efficiency of *B. altitudinis* RSP75 on agricultural wastes depicted its intricate enzyme activity, creating pores and causing deconstruction of the cellulose. The FESEM imaging of the hydrolyzed biomass together with enzyme assays revealed the inherent capacity of *B. altitudinis* RSP75 to valorize LC bioconversion, thereby warranting its further characterization for possible industrial applications. Similarly, FTIR spectroscopic analysis of the FP suggested the elimination of bands that represent cellulose contents. Further, the mass spectrometric analysis revealed compatibility of the bacterium with a fermentation-efficient yeast strain which also suggests its industrial significance towards biofuel production.

## Figures and Tables

**Table 1:** MALDI-TOF mass spectrometry-based biotyping of bacteria isolated from the gut system of the red flour beetle, *T. castaneum*.

Sr. No.	Isolate Code	Best Hit	MALDI-TOF Score
1	TC5	*Achromobacter spanius*	2.057
2	TC7	*Escherichia hermannii*	1.824
3	TC10	*Bacillus subtilis*	1.946
4	TC11	*Bacillus* sp.	1.806
5	TC16	*Enterococcus faecalis*	1.855
6	TC18	*Citrobacter freundii*	1.851
7	TC24	*Enterococcus faecalis*	2.157
8	TC27	*Achromobacter insolitus*	2.244
9	TC39	*Bacillus subtilis*	1.802
10	TC41	*Kluyvera georgiana*	2.244
11	TC55	*Bacillus* sp.	2.218
12	TC67	*Cronobacter sakazakii*	1.828
13	TC70	*Escherichia coli*	1.883
14	RSP75	*Bacillus* sp.	2.207
15	TC91	*Kluyvera ascorbata*	1.915

**Table 2:** Mass spectrometry-based identification of the fermentation products generated during the co-culture of *B. altitudinis* RSP75 with *Saccharomyces cerevisiae*.

Peak No.	Compound Name	Area (%)	Retention Index	Rt (min)
1	Ethanol	0.78	45	2.523
2	Carbon dioxide	7.39	44	1.490
3	Acetic acid	18.31	576	2.025
4	Acetone alcohol	14.57	698	2.275
5	Acetyldehyde	6.00	29	3.115
6	2-Propanone	1.09	698	2.472

**Table 3:** The comparison of the enzyme activity of *B. altitudinis* RSP75 with some previously reported aerobic Gram-positive cellulose-degrading bacterial strains.

Bacteria	NCBI Accession	Substrate Clearance Zone (mm)	Cellulase Activity IU/mL (or mg) Extract	Xylanase Activity IU/mL Extract	Reference
*B. altitudinis* RSP75	MW559543	28	47.1 ± 3	60.2 ± 2	This study
*B. subtilis* 1AJ3	MG062801	19	0.04	ND	[72]
*B. methylotrophicus* 1EJ7	MG062824	19	0.025	ND	
*Bacillus* sp. JMP-A	HM776393	11	27.10	ND	[73]
*Bacillus* sp. NT4	GU458276	ND	~2.5	ND	[74]
*Bacillus megaterium* RU4	NA	14.5	ND	ND	[75]
*Paenibacillus* sp. C1	NA	NA	0.9	ND	[76]
*Bacillus anthracis* AR426	LN829572	ND	0.36 ± 0.04	ND	[77]
*Cohnella formosensis* AR92	FJ976043	ND	0.15 ± 0.03	ND	
*B. tequilensis* G9	KR866144	25	598.07	74.02	[29]
*Bacillus megaterium* SCMC89	KF358455	>8	ND	ND	[78]
*Exiguobacterium marinum* TN25	NA	16	ND	ND	[79]
*B. subtilis* BY-2	KC414931	NA	1.52	ND	[80]
*Bacillus amyloliquefaciens* SS35	AB679994	NA	0.079	ND	[81]
*Cellulomonas* sp.	NA	NA	0.0336	ND	[82]
*Bacillus* sp.	NA	NA	0.0196	ND	
*Micrococcus* sp.	NA	NA	0.0152	ND	

ND, not detected; NA, not available.

## Data Availability

All the data sets generated for this research are included in the manuscript or Supplementary Files.

## Supplementary Materials

The following are available online at https://www.mdpi.com/article/10.3390/microorganisms9091952/s1, Figure S1: Plate based assay for screening the cellulose degrading activity of the isolated bacteria. (A) Isolates patched and grown on BMS agar plates showing zones of CMC clearance around the bacterial colonies indicative of cellulolytic activity. (B) Percentage (%) of isolates depicting various hydrolytic capacity (HC) ratios on BMS agar media containing 0.5% CMC as carbon source.; Figure S2: A dot plot alignment of the 16S rDNA gene sequence from *B. altitudinis* RSP75 against the same gene sequence of its closest NCBI relative *B. altitudinis* (accession no. MN746197); Figure S3: Enzymatic profile of the *B. altitudinis* RSP75 on various commercial and agro-wastes used as substrates over the period of incubation. (a) Endoglucanase; (b) Cellobiase; (c) Xylanase, and (d) Glucoamylase activities. Values are means ± SD of three or more independent replicates. GS: grass straw; CCP: corn cob powder; SD: sawdust; FP: Filter paper; WH: Wheat husk; SCB: Sugarcane bagasse; Figure S4: The gas chromatography-Mass Spectrometry (GC-MS) analysis for identification of metabolites co-culturing the *B. altitudinis* RSP7 with *Sacharomycescerevisae* for fermentation of reducing sugars produced from wheat husk. Mass spectra of the metabolites produced from fermentation of sugars in culture media A), Chromatogram of the fermentation products obtained from the culture broth B); Table S1: Reports on the isolation of *B. altitudinis* strains from various environments/sources and their characteristics.
