# Supplementary material for: Valorization Potential of a Novel Bacterial Strain, Bacillus altitudinis RSP75, towards Lignocellulose Bioconversion: An Assessment of Symbiotic Bacteria from the Stored Grain Pest, Tribolium castaneum
Source: Microorganisms. 2021 Sep 14;9(9):1952. doi: 10.3390/microorganisms9091952 (PMC8468446; doi:10.3390/microorganisms9091952)
Supplement: Supplementary file 1 [file microorganisms-09-01952-s001.zip › microorganisms-1358777-supplementary.pdf]

Supplementary Information

For

Valorization potential of a novel bacterial strain, *Bacillus altitudinis* RSP75, towards lignocellulose bioconversion: An assessment on symbiotic bacteria from the stored grain pest, *Tribolium castaneum*

Journal: Microorganisms

Mudasir A. Dar[1,2], Neeraja P. Dhole[2#], Rongrong Xie[1], Kiran D. Pawar[3], Kalim Ullah[4], Praveen Rahi[5], Radhakrishna S. Pandit[2*], Jianzhong Sun[1*]

1. Biofuels Institute, School of the Environment and Safety Engineering, Jiangsu University, Zhenjiang-212013, China.
2. Department of Zoology, Savitribai Phule Pune University, Ganeshkhind, Pune, Maharashtra-411007, India.
3. School of Nanoscience and Biotechnology, Shivaji University, Vidyanagar, Kolhapur, Maharashtra-416004, India.
4. School of Medicine, Jiangsu University, Zhenjiang-212013, China.
5. National Centre for Microbial Research, Trinity Complex, Pashan Pune, Maharashtra- 411021, India.

*__Correspondence:__

Radhakrishna S. Pandit: Ganeshkhind, Pune, Maharashtra- 411007, India.

(Tel.: +91 (0) 2025601436, fax.: +91 (0) 2025690617), mudasir.dar@unipune.ac.in

panditrao499@gmail.com

Jianzhong Sun: Zhenjiang, Jiangsu Province-212013, China.

(Tel.: +86 15052919625, fax.: +86 0511-88790955) jzsun1002@ujs.edu.cn

#Co-first author

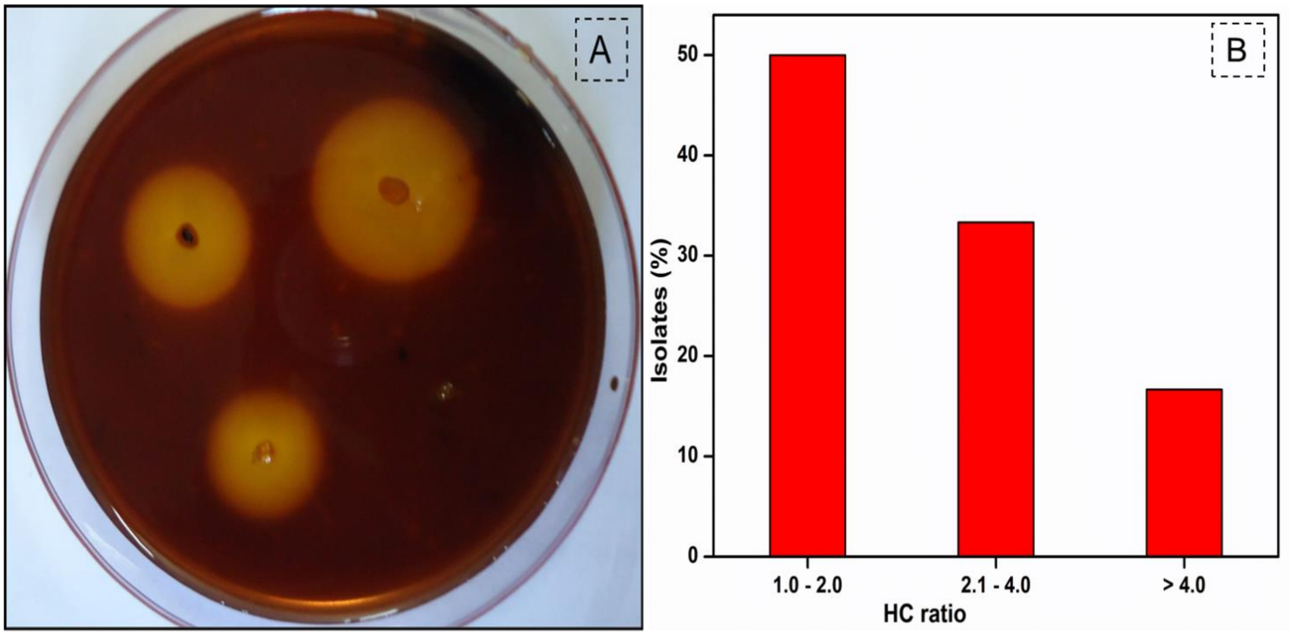

Fig. S1. Plate based assay for screening the cellulose degrading activity of the isolated bacteria. A) Isolates patched and grown on BMS agar plates showing zones of CMC clearance around the bacterial colonies' indicative of cellulolytic activity. B) Percentage (%) of isolates depicting various hydrolytic capacity (HC) ratios on BMS agar media containing 0.5% CMC as carbon source.

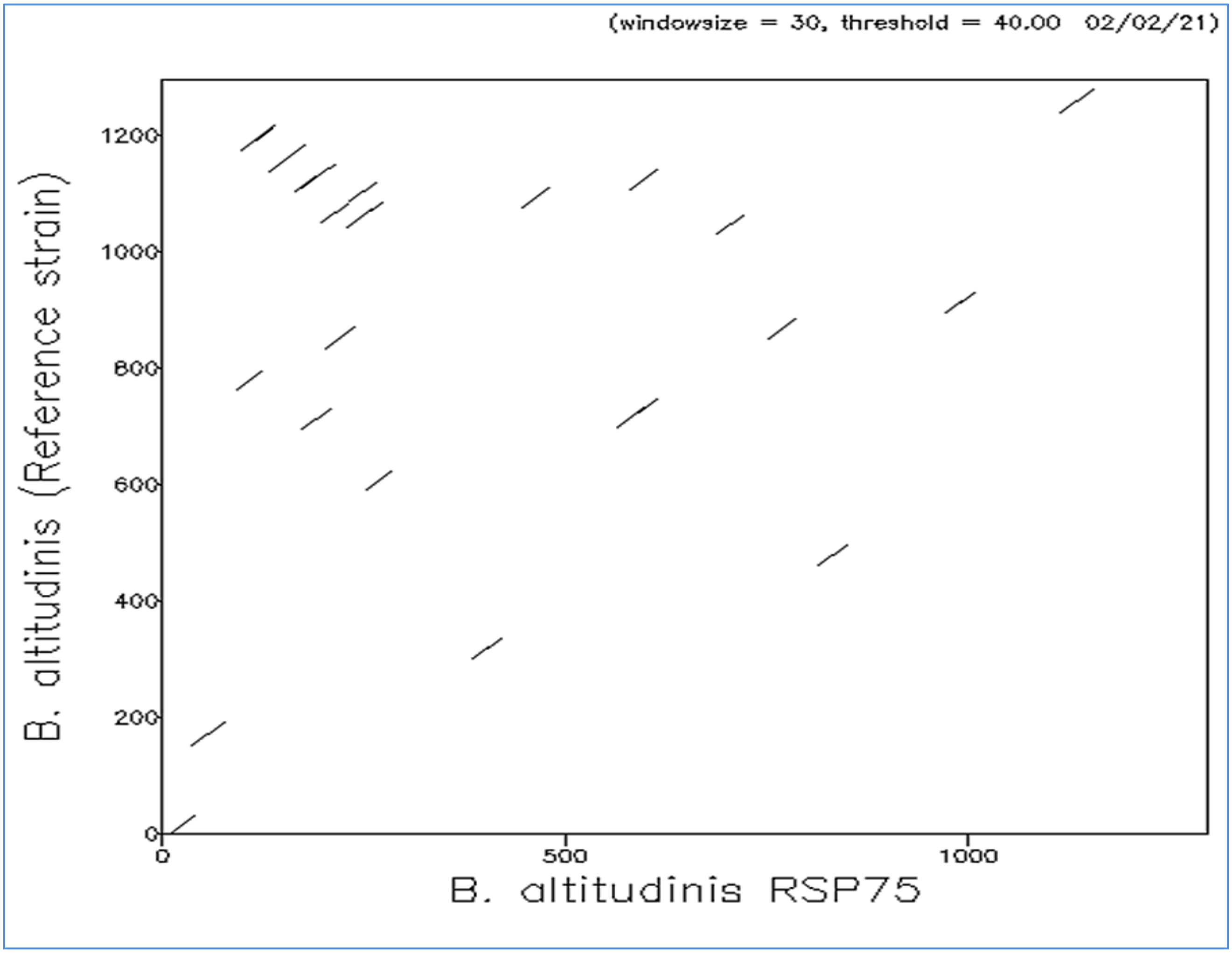

Fig. S2. A dot plot alignment of the 16S rDNA gene sequence from *B. altitudinis* RSP75 against the same gene sequence of its closest NCBI relative *B. altitudinis* LXJ69 (accession no. MN746197).

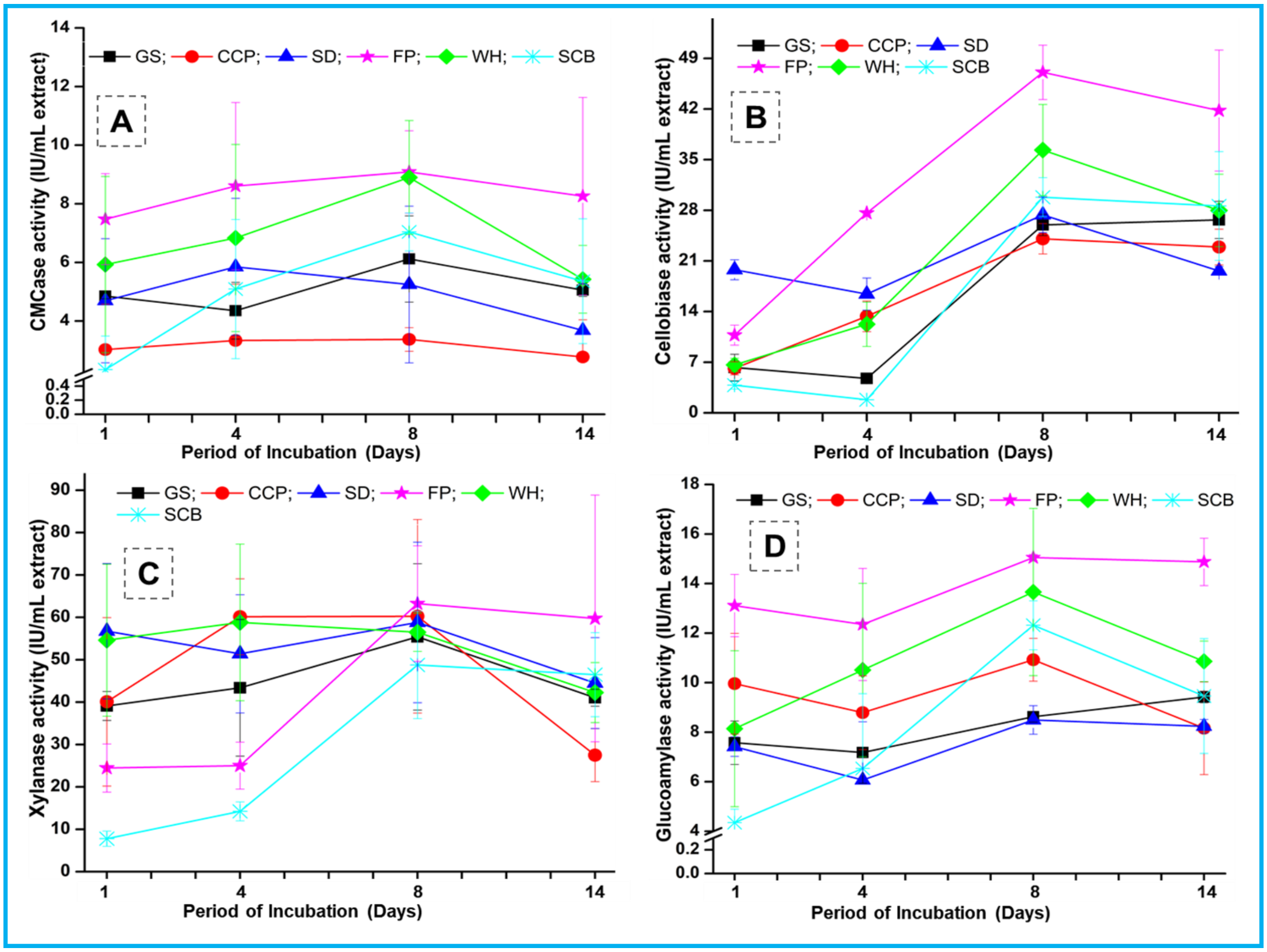

Fig. S3. Enzymatic profile of the *B. altitudinis* RSP75 on various commercial and agro-wastes used as substrates over the period of incubation. a) Endoglucanase; b) Cellobiase; c) Xylanase, and d) Glucoamylase activities. Values are means ± SD of three or more independent replicates. GS: grass straw; CCP: corn cob powder; SD: sawdust; FP: Filter paper; WH: Wheat husk; SCB: Sugarcane bagasse.

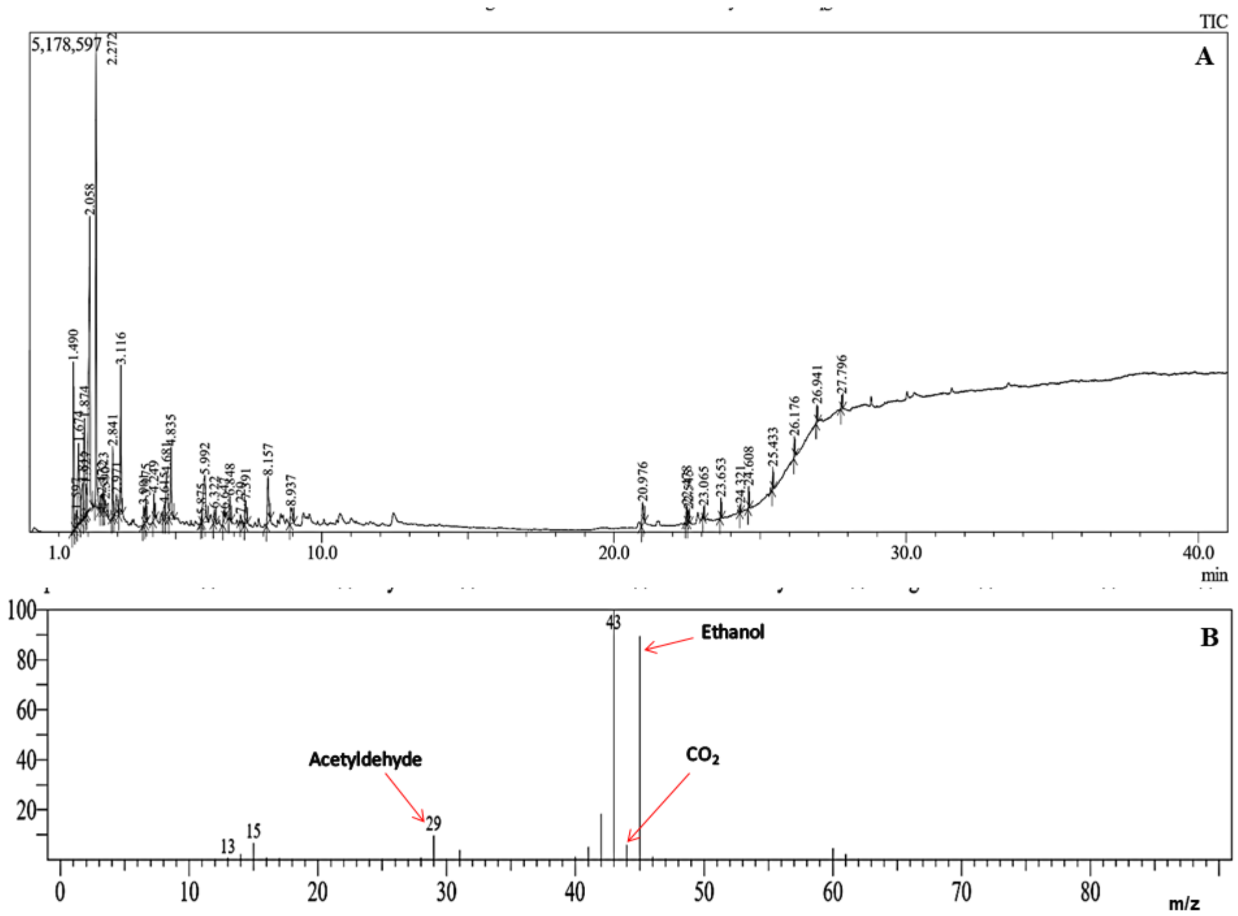

Fig. S4. The gas chromatography-Mass Spectrometry (GC-MS) analysis for identification of metabolites co-culturing the *B. altitudinis* RSP7 with *Sacharomycescerevisae* for fermentation of reducing sugars produced from wheat husk. Mass spectra of the metabolites produced from fermentation of sugars in culture media A), Chromatogram of the fermentation products obtained from the culture broth B).

Table S1 Reports on the isolation of *B. altitudinis* strains from various environments/sources and their characteristics.

Sr. no.	Strain	NCBI accession	Isolation Source	Method of isolation	Purpose of study	Reference
1	*Bacillus altitudinis* RSP75	MW559543	Beetle	16S rRNA	Cellulose degradation	This study
2	*B. altitudinis* AP-MSU	. HM582688	Fish	16S rRNA	Esterase production	[1]
3	*B. altitudinis* APS MSU	ND	Fish	16S rRNA	Cellulase production	[2]
4	*B. altitudinis*SGAir0031	CP022319, CP022320	Air samples	Genomics	Identification/ Bioprospection	[3]
5	*B. altitudinis*41KF2b^T	AJ831842	Air samples	16S rRNA	Bioprospection	[4]
6	*B. altitudinis* PAE4	PRJEB26379	Coastal ridge	Genomics	Plant -growth promoter	[5]
7	*B. altitudinis* AMCC 101304	ND	Rhizosphere soil	16S rRNA	Antibacterial activity	[6]
8	*B. altitudinis* DSMZ 26896 (earlier *B. invictae* Bi.FFUP1^T)	JXAI00000000	Health product	Genomics	Taxonomic classification	[7]
9	*B. altitudinis* AB4/AB6	KF640213, KF640212	Apple	16S rRNA	Disease causing agent	[8]
10	*B. altitudinis* FD48	CP025643	Rice phylloplane	Genomics	Plant -growth promoter	[9]
11	*B. altitudinis* P-10	CP024204	Rhizosphere of rice	Genomics	Antibacterial activity	[10]
12	*B. altitudinis* WR10	KY416926	Wheat	16S rRNA	IAA production	[11]
13	*B. altitudinis* YC-9	JX869996	Silt	16S rRNA	Gene cloning	[12]
14	*B. altitudinis* GVC11	FN668692	Soil	16S rRNA	Dehairing	[13]
15	*B. altitudinis* BRHS/S-73	JF899300	Rhizosphere	16S rRNA	Antibacterial activity	[14]

ND: not defined, IAA: indole 3, acetic acid

References

1. Esakkiraj, P.; Usha, R.; Palavesam, A.; Immanuel, G. Solid-state production of esterase using fish processing wastes by *Bacillus altitudinis* AP-MSU. *Food Bioprod. Process.* **2012**, *90*, 370–376, https://doi.org/10.1016/j.fbp.2011.12.008.

2. Sreeja, S.J.; Jeba-Malar, P.W.; Sharmila-Joseph, F.R.; Tiburcius, S.; Immanuel, G.; Palavesam, A. Optimization of cellulase production by *Bacillus altitudinis* APS MSU and *Bacillus licheniformis* APS2 MSU, gut isolates of fish *Etroplus suratensis*. *Int. J. Adv. Res. Technol.* **2013**, *2*, 401–406.
3. Vettath, V.K.; Junqueira, A.C.M.; Uchida, A.; Purbojati, R.W.; Houghton, J.N.I.; Chénard, C.; Drautz-Moses, D.I.; Wong, A.; Kolundžija, S.; Clare, M.E.; et al. Complete genome sequence of *Bacillus altitudinis* type strain SGAir0031 isolated from tropical air collected in Singapore. *Genome Announc*. 2017, 5, e01260-17, https://doi.org/10.1128/genomeA.01260-17.
4. Shivaji, S.; Chaturvedi, P.; Suresh, K.; Reddy, G.S.N.; Dutt, C.B.S.; Wainwright, M.; Narlikar, J.V.; Bhargava, P.M. *Bacillus Aerius* sp. nov., *Bacillus aerophilus* sp. nov., *Bacillus stratosphericus* sp. nov. and *Bacillus altitudinis* sp. nov., isolated from cryogenic tubes used for collecting air samples from high altitudes. *Int. J. Syst. Evol. Microbiol.* **2006**, *56*, 1465–1473, http://dx.doi.org/10.1099/ijs.0.64029-0.
5. Ibrahim, N.A.G.A.A.; Omar, M.N.A.M.; El-Heba, G.A.A.; Moënne-Loccoz, Y.; PrigentCombaret, C.; Muller, D. Draft genome sequence of plant growth-promoting *Bacillus altitudinis* strain PAE4. *Microbiol. Resour. Announc.* **2018**, *7*, e00962-18, https://doi.org/10.1128/MRA.00962-18.
6. Li, B.; Wang, B.; Pan, P.; Li, P.; Qi, Z.; Zhang, Q.; Shi, C.; Hao, W.; Zhou, B.; Lin, R. *Bacillus altitudinis* strain AMCC 2019, 101304, a novel potential biocontrol agent for potato common scab. *Biocontrol Sci. Technol.* **2019**, *29*, 1009–1022, https://doi.org/10.1080/09583157.2019.1641791.
7. Liu, Y.; Du, J.; Shao, Z.; Lai, Q. Reclassification of *Bacillus invictae* as a later heterotypic synonym of *Bacillus altitudinis*. *Int. J. Syst. Evol. Microbiol.* **2015**, *65*, 2769–2773, https://doi.org/10.1099/ijs.0.000336.
8. Elbanna, K.; Elnaggar, S.; Bakeer, A. Characterization of *Bacillus altitudinisas* a new causative agent of bacterial soft rot. *J. Phytopathol.* **2014**, *162*, 712–722, https://doi.org/10.1111/jph.12250.
9. Kumaravel, S.; Thankappan, S.; Raghupathi, S.; Uthandi, S. Draft genome sequence of plant growth-promoting and drought-tolerant *Bacillus altitudinis* FD48, isolated from rice phylloplane. *Genome Announc.* **2018**, *6*, 00019-18, https://doi.org/10.1128/genomeA.00019-18.
10. Budiharjo, A.; Jeong, H.; Wulandari, D.; Lee, S.; Ryu, C.M. Complete genome sequence of *Bacillus altitudinis* P-10, a potential bioprotectant against *Xanthomonas oryzae* pv. oryzae, isolated from rice rhizosphere in Java, Indonesia. *Genome Announc.* **2017**, *5*, e01388-17, https://doi.org/10.1128/genomeA.01388-17.
11. Sun, Z.; Liu, K.; Zhang, J.; Zhang, Y.; Xu, K.; Yu, D.; Wang, J.; Hu, L.; Chen, L.; Li, C. IAA producing *Bacillus altitudinis* alleviates iron stress in *Triticum aestivum* L. seedling by both bioleaching of iron and up-regulation of genes encoding ferritins. *Plant Soil* **2017**, *419*, 1–11, https://doi.org/10.1007/s11104-017-3218-9.
12. Mao, S.; Lu, Z.; Zhang, C.; Lu, F.; Bie, X. Purification, characterization, and heterologous expression of a thermostable β-1,3-1,4-glucanase from *Bacillus altitudinis* YC-9. *Appl. Biochem. Biotechnol.* **2013**, *169*, 960–975, http://dx.doi.org/10.1007/s12010-012-0064-3.
13. Kumar, E.V.; Srijana, M.; Kiran Kumar, K.; Harikrishna, N.; Reddy, G. A novel serine alkaline protease from *Bacillus altitudinis* GVC11 and its application as a dehairing agent. *Bioprocess Biosyst. Eng.* **2011**, *34*, 403–409, https://doi.org/10.1007/s00449-010-0483-x.
14. Sunar, K.; Dey, P.; Chakraborty, U.; Chakraborty, B. Biocontrol efficacy and plant growth promoting activity of *Bacillus altitudinis* isolated from Darjeeling hills, India. *J. Basic Microbiol.* **2013**, *55*, 91–104, https://doi.org/10.1002/jobm.201300227.
